# Impact of the Coronavirus Disease 2019 Pandemic on Dental Visits in Japan

**DOI:** 10.3290/j.ohpd.b4100953

**Published:** 2023-05-17

**Authors:** Ichizo Morita, Shigemitsu Sakuma, Kanae Kondo

**Affiliations:** a Professor of Epidemiology and Statistics, Japanese Red Cross Toyota College of Nursing, Toyota, Aichi, Japan. Study conceptualisation, statistical analysis, wrote the original draft of the manuscript.; b Lecturer, Department of Prosthodontics and Oral Implantology, Aichi Gakuin University, Nagoya, Aichi, Japan. Study conceptualisation, read and approved the manuscript.; c Assistant Professor, Gerontological Nursing, Japanese Red Cross Toyota College of Nursing, Toyota, Aichi, Japan. Study conceptualisation, read and approved the manuscript.

**Keywords:** clinic visits, elderly, interrupted time-series analysis, stay at home orders, universal health insurance

## Abstract

**Purpose:**

This study aimed to clarify the impact of the coronavirus disease 2019 (COVID-19) pandemic on individual dental-visit behaviour and examine the difference between elderly and other individuals regarding the impact on dental visits.

**Materials and Methods:**

An interrupted time-series analysis was performed to examine the change in data from the national database before and after the first declaration of a state of emergency.

**Results:**

The number of patients visiting a dental clinic (NPVDC), number of dental treatment days (NDTD) and dental expenses (DE) during the first declaration of a state of emergency decreased by 22.1%, 17.9%, and 12.5% in the group under 64 years of age and 26.1%, 26.3%, and 20.1% in the group over 65 years of age, respectively, compared with those in the same month of the previous year. Between March and June 2020, the monthly NPVDC and NDTD were significantly reduced (p < 0.001, p = 0.013) in those over 65 years of age. The DE did not change statistically significantly in either the under 64 group or the over 65 group. There was no statistically significant change in the slope of the regression line in the NPVDC, NDTD, and DE before and after the first state-of-emergency declaration.

**Conclusion:**

The first state of emergency greatly reduced the NPVDC, NDTD, and DE compared to those in the previous year. In people aged over 65 years, it might still be unresolved 2 years after the postponement of dental treatment owing to the first declaration of a state of emergency.

In January 2020, the first case of severe acute respiratory syndrome coronavirus 2 (SARS-CoV-2) infection was reported in Japan. Thereafter, the number of patients with coronavirus disease 2019 (COVID-19) increased dramatically. Thus, to control the spread of infection, the government declared a nationwide state of emergency in mid-April 2020. The declaration of a state of emergency called for individuals to refrain from non-essential outings, thereby restricting citizens’ movements. Movement restrictions in Japan have been lenient and not accompanied by penalties, unlike lockdowns. Moreover, medical institutions, including dental clinics, were not considered non-essential outings.^19^ However, some reports have shown that individuals postponed their dental visits.^9,10,13,19,22^ The COVID-19 pandemic has caused changes in dental visit behaviour not only in Japan but also in many other countries.^5,7,11,20,21^

Aging has been recognised as a prominent risk factor for severe disease and death from COVID-19 since the early days of the pandemic.^3,7^ Therefore, older individuals may have refrained from visiting dentists during the COVID-19 pandemic. However, some attitude surveys in Japan point out that elderly people may not particularly refrain from visiting dentists compared to the younger generation. A few studies in other countries reported that elderly individuals were less likely to refrain from dental visits than younger individuals,^5,11^ while others reported that elderly individuals refrained from visiting dentists.^4,20^

There have been fragmentary reports of the impact of declaring a state of emergency and subsequent COVID-19 pandemic on people’s dental visit behaviour. However, no reports have quantitatively evaluated the impact of the COVID-19 pandemic based on nationwide dental-visit information. The COVID-19 pandemic is expected to continue, with a possibility that similar new infectious diseases will spread in the future. Therefore, clarifying the impact of the pandemic on dental visits will contribute to future dental-care policies.

This study aimed to clarify the impact of the COVID-19 pandemic on people’s dental-visit behaviour in Japan. Moreover, this study examined the difference between elderly and other individuals regarding the impact on dental visits.

## Materials and Methods

### Data Collection

Data from the medical insurance medical expense database were used for the analysis.^16^ This database is part of the survey on the trend of medical care expenditures conducted by the Ministry of Health, Labor, and Welfare and provides information nationwide from April 1984 to February 2022 (as of August 2022). The health insurance medical expense database collects information on the number of medical- and dental-visit patients, number of treatment days, and medical and dental expenses based on the medical and dental care provided through the universal health insurance system (98.9% population coverage). The number of patients visiting a dental clinic (NPVDC) is the total number of individuals who received dental care at least once per month at each medical clinic nationally. The number of dental treatment days (NDTD) is defined as the total number of days a patient received dental care in a month nationally. Dental expenses (DE) is the amount of dental care per month nationally in yens.

This study was a secondary analysis of the data and was approved by the Ethics Committee of the Japanese Red Cross Toyota College of Nursing (project no. 2211).

### Statistical Methods

An interrupted time-series analysis^1^ was performed to examine the changes in the NPVDC, NDTD, and DE before and after the first declaration of a state of emergency. Analyses were performed and divided into patients aged 64 years and below (under 64 group) and those aged 65 years and above (over 65 group). The data period used for the analysis was from April 2015 to February 2022.

Furthermore, changes were calculated in the NPVDC, NDTD, and DE in June 2020, immediately after the state of emergency was declared. Changes in the NPVDC, NDTD, and DE were calculated as the percentage of change of the value for June 2020 obtained using the regression equation from June 2020 to February 2022 to the estimated value for June 2020 obtained using the regression equation from April 2015 to March 2020.

Analyses were performed using IBM SPSS version 28 (IBM; Armonk, NY, USA).

## Results

### Impact of the First Declaration of a State of Emergency

**Fig 1:**
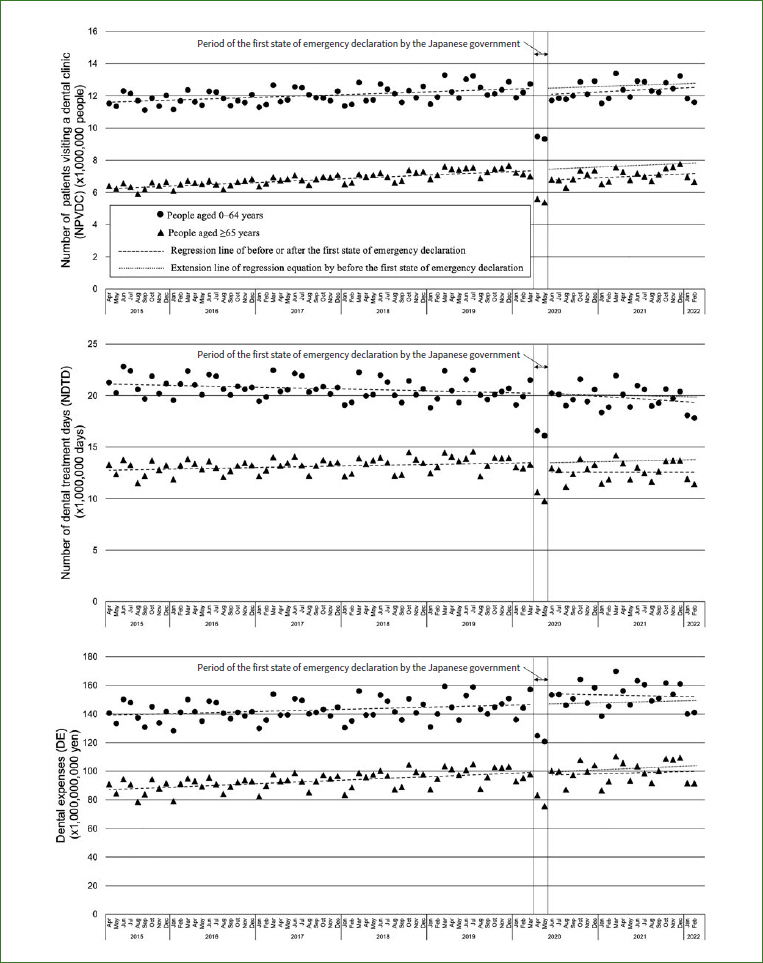
Trend changes in the number of patients visiting a dental clinic, treatment days, and expenses before and after the first state of emergency declaration.

In April and May 2000, the government declared the first state of emergency; the NPVDC, NDTD, and DE decreased from corresponding values in the previous month (Fig 1). Comparing these 2 months with the average values for the same month of the previous year, in the under 64 and over 65 groups, the NPVDC, NDTD, and DE showed changes of -22.1%, -17.9%, and -12.5% (under 64) and -26.1%, -26.3%, and -20.1% (over 65). In April and May 2000, the first declaration of a state of emergency required people to refrain from non-essential outings, and the values of the NPVDC, NDTD, and DE became particularly irregular. Therefore, an interrupted time-series analysis was conducted to focus on before and after the declaration of the state of emergency, excluding April and May 2000.

### Impact of the COVID-19 Pandemic on the NPVDC

The monthly NPVDC showed a statistically significant increase (p < 0.001) in both the under 64 and over 65 groups until March 2020, before the state of emergency was declared (Table 1). Between March and June 2020, there was no statistically significant level change (p = 0.135) among those under 64, but a statistically significant decrease (p < 0.001) was observed in those over 65. The June 2020 estimate based on regression equations before and after the state of emergency was declared showed a 3.2% decrease in the NPVDC for those under 64 and a 9.1% decrease for those over 65 (Table 2). There was no statistically significant change in the slope of the regression line in the NPVDC before and after the declaration of the state of emergency.

**Table 1:** Parameter estimates, 95% confidence intervals, and p-values from segmented regression model describing the trend of the monthly number of patients visiting a dental clinic, treatment days, and expenses

		Coefficient	95% confidence interval		p-value^a^
			Lower limit	Upper limit	
**People aged 0–64 years**					
Number of patients visiting a dental clinic (NPVDC; unit: one thousand patients)	Intercept	12466.0	12215.4	12716.6	<0.001
	Baseline trend	14.7	7.6	21.9	<0.001
	Level change	-361.0	-836.2	114.3	0.135
	Slope change	6.0	-29.3	41.3	0.736
Number of dental treatment days (NDTD; unit: one thousand days)	Intercept	20256.1	19726.8	20785.5	<0.001
	Baseline trend	-14.7	-29.8	0.4	0.056
	Level change	-23.2	-1027.1	980.6	0.963
	Slope change	-31.8	-106.3	42.7	0.399
Dental expenses (DE; unit: 1 million yen)	Intercept	146551.5	142601.2	150501.9	<0.001
	Baseline trend	125.8	13.2	238.4	0.029
	Level change	7173.9	-317.6	14665.3	0.060
	Slope change	-207.3	-763.3	348.8	0.460
**People aged ≥65 years**					
Number of patients visiting a dental clinic (NPVDC; unit: one thousand patients)	Intercept	7435.0	7291.3	7578.6	<0.001
	Baseline trend	19.6	15.5	23.7	<0.001
	Level change	-625.0	-897.4	-352.5	<0.001
	Slope change	2.7	-17.5	22.9	0.792
Number of dental treatment days (NDTD; unit: one thousand days)	Intercept	13596.7	13211.9	13981.6	<0.001
	Baseline trend	12.6	1.6	23.6	0.025
	Level change	-929.7	-1659.5	-200.0	0.013
	Slope change	-12.5	-66.7	41.7	0.647
Dental expenses (DE; unit: 1 million yen)	Intercept	99532.3	96489.0	102575.5	<0.001
	Baseline trend	201.5	114.7	288.3	<0.001
	Level change	-1322.7	-7093.9	4448.4	0.649
	Slope change	-80.8	-509.1	347.5	0.708

The model describes the trends and changes in the error rate during the pre-implementation, implementation, and post-implementation phases. ^a^Calculated using Student’s t-test. Baseline trend: Trend from April 2015 to March 2020. Level change: level change from March 2020 to June 2020. Slope change: slope change from before March 2020 to after June 2020.

**Table 2:** Changes in the NPVDC, NDTD, and DE of the value for June 2020 obtained using the regression equation from before and after the first declaration of a state of emergency

	People aged 0–64 years			People aged ≥65 years		
	From regression equation before declaration^a^	From regression equation after declaration^b^	Change^c^	From regression equation before declaration^a^	From regression equation after declaration^b^	Change^c^
NPVDC	12510.2	12105.0	- 3.2	7493.7	6810.0	- 9.1
NDTD	20212.0	20232.9	0.1	13634.5	12667.0	- 7.1
DE	146929.0	153725.4	4.6	100136.8	98209.5	- 1.9

NPVDC: number of patients visiting a dental clinic (unit: thousand patients); NDTD: number of dental treatment days (unit: thousand days); DE: dental expenses (unit: million yen). ^a^From April 2015 to March 2020; ^b^from June 2020 to February 2022; ^c^percentage of change = (from regression equation after declaration/from regression equation before declaration – 1 ) x 100 (%).

### Impact of the COVID-19 Pandemic on the NDTD

The monthly NDTD showed a decreasing trend (p = 0.056) in the under-64 group and a statistically significant increase (p = 0.025) in the over-65 group until March 2020, before the state of emergency was declared. Between March and June 2020, there was no statistically significant level change (p = 0.963) in those under 64, but a statistically significant level decrease (p = 0.013) was observed in those over 65 years of age. The June 2020 estimate based on regression equations before and after the state of emergency was declared showed a 0.1% increase in the NDTD for those under 64 and a 7.1% decrease for those over 65. There was no statistically significant change in the slope of the regression line in the NDTD before and after the declaration of the state of emergency.

### Impact of the COVID-19 Pandemic on the DE

Monthly DE showed a statistically significant increase in both the under-64 (p = 0.029) and over-65 groups (p < 0.001) until March 2020, before the state of emergency was declared. Between March and June 2020, there was no statistically significant level change in either the under-64 (p = 0.060) or the over- 65 groups (p = 0.649). The June 2020 estimate based on regression equations before and after the declaration of a state of emergency showed a 4.6% level increase in DE for those under 64 and a 1.9% decrease for those over 65. There was no statistically significant change in the slope of the regression line for DE before and after the declaration of a state of emergency.

## Discussion

The study results demonstrated that the first declaration of a state of emergency resulted in a statistically significant reduction in dental visits in Japan. Moreover, it showed a recovery once the state of emergency was lifted. However, for patients over 65 years of age, a statistically significant change was observed in the regression line for both the NPVDC and NDTD, indicating that the influence of the reduction in dental visits continued even after the declaration of a state of emergency.

In Japan, the first case of SARS-CoV-2 infection was reported in January 2020 and a cluster outbreak was confirmed in February. At the end of February 2020, elementary and junior high schools across the country were ordered to shut, and in Hokkaido prefecture, where the spread of infection was notable, a state of emergency was declared, and people were asked to refrain from going out on weekends. In March, individuals in major cities were asked to refrain from going out on weekends. On April 7, 2020, the government declared a state of emergency in seven prefectures, which was expanded to all prefectures on April 16. In Japan, lockdowns such as city blockades and forced curfews were not implemented; only outdoor visits were curbed and contact between individuals was reduced to a feasible extent. However, the declaration of a state of emergency brought an approximately 80% reduction in the flow of people in central Tokyo, and an effect was observed. The target area of the first declaration of a state of emergency decreased on May 14 and 21, 2020, and it was lifted entirely on May 25 of that year.

On January 8, 2021, the second state of emergency was declared in Tokyo and the surrounding three prefectures and lifted on March 21, 2021. However, on April 25, 2021, the third state of emergency was declared, which was lifted on June 20, 2021. The fourth state of emergency was declared from July 12, 2021 to September 30, 2021.

Additionally, some prefectures applied a quasi-state of emergency from April 5, 2021 to September 30, 2021, and from January 9, 2022 to March 21, 2022. Both the declaration of a state of emergency and quasi-state of emergency involved requiring people to refrain from going out, limiting the use of public facilities, requesting businesses to suspend operations, requesting/ordering shortened business hours of restaurants, and requesting or ordering restrictions on the holding of events. These restrictions were issued by the Japanese government during the period covered in this study.

The results of this study show that the impact of the first state of emergency declaration was remarkable; however, the impact of the second and subsequent state of emergency declarations was much less than that of the first. In the early days of the COVID-19 pandemic, governments and dental associations world wide issued statements recommending refraining from visiting dentists, except in emergencies.^6,8,11,14,15,20^ In Japan, the Ministry of Health, Labor, and Welfare only notified medical institutions of thorough measures to prevent nosocomial infections. In April, immediately after the first state of emergency was declared, the Japan Dental Association issued a message to the public urging people to postpone dental visits. Subsequently, in May, the Japan Dental Association encouraged individuals to consider the risks and benefits of dental visits when making a decision. Additionally, the Japan Dental Association published countermeasure guidelines for dental clinics and strove to prevent infections caused by dental treatments. The fact that the Japan Dental Association presented a clear policy on dental visits and a vision for infection control may be one of the factors behind the recovery in dental visits since June 2020.

In April and May 2020, when the first state of emergency was declared, the NPVDC, NDTD, and DE greatly decreased in Japan. From January to March 2020, compared to the same period in 2019, the total number of dental visits decreased by 5.1% in Taiwan, and the numbers of emergency periodontal treatments, tooth scaling, and periodontal surgeries decreased statistically significantly.^12^ However, restorations due to tooth decay, root canal treatment, emergency endodontic treatment, and other dental treatments did not show any statistically significant changes. In Saudi Arabia, more than half of the population was hesitant to visit a dentist, even when they experienced pain from periodontitis or pulpitis during the COVID-19 pandemic.^14^ In Buenos Aires in 2020, the rate of emergency dental treatment showed a five-fold reduction compared with 2019.^20^

Dental treatments using handpieces, three-way syringes, and ultrasonic scalers generate aerosols that contain saliva.^6^ Aerosols are known to transmit SARS-CoV-2, making people hesitant to visit dentists.^6,14^ Another reason for the decrease in dental visits is the restriction of dental care owing to the risk of infection to the dental staff themselves.^6^

Several studies have reported that age was not statistically significantly associated with postponing dental attendance in Japanese individuals, according to an online survey.^9,19,22^ The results of this study showed a significant decrease in the NPVDC and NDTD before and after the first state of emergency in people over 65 years of age; however, no statistically significant decrease was observed in people younger than 64 years.

The association between dental visits and age in other countries varies, and a study from Saudi Arabia did not include an age variable as a predictor of emergency dental visits during the COVID-19 pandemic^14^ In contrast, studies in the United States and Germany have reported that age over 65 years was not a factor in delaying dental visits.^5,11^ Conversely, reports from Spain and Argentina indicated that being elderly was a factor in refraining from dental visits.^4,20^ Many reports were biased toward sampling from online surveys and were based on local data. Additionally, people’s responses differed depending on whether there were restrictions on their activities during the period covered by the survey. A survey based on mobile phone location information revealed that during the first state of emergency declaration in Japan, elderly people were less likely to refrain from going out than young individuals.^23^ Behavioural restraint of elderly people may be more pronounced in dental visits than in other behaviours.

The policies of each country and region affect people’s behaviour. In Japan, approximately 104,000 dentists and 132,000 dental hygienists are employed at more than 86,000 dental practices. Most dental practices are run privately for profit. However, medical and dental care is provided by universal health insurance systems organised by the Japanese government,^18^ and access to medical and dental care is unlimited. The unit price for medical and dental care is determined by the government. Individuals have to pay 10%–30% of their medical and dental expenses, with the rest covered by insurance. In this context, the results of this study covered the whole of Japan and demonstrated people’s dental visit behaviour in the absence of expensive dental treatment. Evaluating changes in people’s behaviour during the COVID-19 pandemic under different conditions requires more research.

Unexpectedly, although not statistically significantly, DE increased after the declaration of a state of emergency was lifted compared to that before the declaration in people under 64 years of age. It was somewhat surprising that medical expenses increased despite no increase in the NPVDC and NDTD. In the universal health insurance system, the remuneration unit price is determined for each dental treatment. Therefore, treatments that were postponed owing to the state of emergency may have been added after the declaration was lifted. These added treatments may contribute to the relief of the patient’s deferred treatment needs. In the near future, the regression line after the declaration is lifted will fall below the extrapolation of the regression line before the declaration. Approximately 2 years were required to recover from the increase in dental treatment needs caused by the postponement of dental treatment owing to the first declaration of the state of emergency for two months.

In the over-65 group, no DE increase was observed after the state of emergency was lifted, which differs from the under-64 group. The postponement of dental treatment demand may still not be resolved owing to the first declaration of a state of emergency in the over-65 group. Therefore, countermeasures against the loss of opportunity to receive dental treatment in elderly people are required.

An important limitation of this study is that it did not include private DE. Patients can pay for private treatment if they request a qualitatively higher alternative of care not covered by the universal health insurance system. Its share in dentistry is approximately 20% on a monetary basis;^2^ this study did not include these practices. Finally, only 22 months were available for evaluation after the first declaration of a state of emergency. In Japan, COVID-19 was downgraded on 8 May 2023 to the status of seasonal flu, so that people’s behaviour is returning to normal, but the long-term impact of the COVID-19 pandemic must be ascertained.

## Conclusion

In the first state of emergency, the NPVDC, NDTD, and DE were reduced by >10% compared with those in the previous year in Japan. The increased DE for people aged under 64 years of age after the first declaration of a state of emergency may have contributed to the resolution of the demand for dental treatment that was postponed during the declaration period. However, in people older than 65 years, it might still be unresolved 2 years after the postponement of dental treatment demand owing to the first declaration of a state of emergency.
